# The microbiota and the host organism switch between cooperation and competition based on dietary iron levels

**DOI:** 10.1080/19490976.2024.2361660

**Published:** 2024-06-27

**Authors:** Marie-Louise Noordine, Yohannes Seyoum, Aurélia Bruneau, Kaleab Baye, Thibaud Lefebvre, Claire Cherbuy, François Canonne-Hergaux, Gaël Nicolas, Christèle Humblot, Muriel Thomas

**Affiliations:** a Micalis Institute, Institut national de recherche pour l’agriculture, l’alimentation et l’environnement (INRAE), AgroParisTech, Université Paris-Saclay, UMR1319, Jouy-en-Josas, France; b Center for Microbiome Medicine (PaCeMM) FHU, AP-HP, Paris, Ile-de-France, France; c Center for Food Science and Nutrition, College of Natural and Computational Sciences, Addis Ababa University, Addis Ababa, Ethiopia; d QualiSud, Université de Montpellier, Avignon Université, CIRAD, Institut Agro, IRD, Université de la Réunion, Montpellier Cedex, France; e Assistance Publique-Hôpitaux de Paris, Centre Français des Porphyries, Hôpital Louis Mourier, Colombes, France; f Institut National de la Santé et de la Recherche Médicale, U1149, Centre de Recherches sur l’Inflammation, Paris, France; g IRSD, Université de Toulouse, INSERM, INRAE, ENVT, Univ Toulouse III - Paul Sabatier (UPS), Toulouse, France; h U1188 DéTROI, Université de La Réunion, Paris, France; i Université Paris Diderot, site Bichat, Sorbonne Paris Cité, Paris, Ile-de-France, France

**Keywords:** Microbiota, ferritin, Dmt1, splanchnic area, lung, iron parameters

## Abstract

The microbiota significantly impacts digestive epithelium functionality, especially in nutrient processing. Given the importance of iron for both the host and the microbiota, we hypothesized that host-microbiota interactions fluctuate with dietary iron levels. We compared germ-free (GF) and conventional mice (SPF) fed iron-containing (65 mg/Kg) or iron-depleted (<6 mg/Kg) diets. The efficacy of iron privation was validated by iron blood parameters. Ferritin and Dmt1, which represent cellular iron storage and transport respectively, were studied in tissues where they are abundant: the duodenum, liver and lung. When the mice were fed an iron-rich diet, the microbiota increased blood hemoglobin and hepcidin and the intestinal ferritin levels, suggesting that the microbiota helps iron storage. When iron was limiting, the microbiota inhibited the expression of the intestinal Dmt1 transporter, likely via the pathway triggered by Hif-2α. The microbiota assists the host in storing intestinal iron when it is abundant and competes with the host by inhibiting Dmt1 in conditions of iron scarcity. Comparison between duodenum, liver and lung indicates organ-specific responses to microbiota and iron availability. Iron depletion induced temporal changes in microbiota composition and activity, reduced α-diversity of microbiota, and led to *Lactobacillaceae* becoming particularly more abundant after 60 days of privation. By inoculating GF mice with a simplified bacterial mixture, we show that the iron-depleted host favors the gut fitness of *Bifidobacterium longum*.

## Introduction

Human beings are holobionts, meaning that they rely on an obligatory co-existence with microbiota.^1–3^ Most of these microbial cells live in the gastrointestinal tract and are responsible for tasks such as digestion, protection through the barrier effect, and the maturation of immunity.^4^ The microbiota connects the gut with other organs, such as the brain, lungs, liver, and muscles through various metabolic and structural signals.^5–7^ In humans, gut bacteria are dominated by five bacterial phyla (*Bacillota, Bacteroidota, Actinomycetota, Pseudomonadota*, and *Verrucomicrobia*), of which the relative abundance and activity are mainly influenced by the environment,^8^ especially dietary intake.^9,10^

Both the microbiota and eukaryotic cells need iron to live, rendering iron central in host-microbe and microbe-microbe interactions.^11–13^ Multiple animal and human studies have examined the effect of iron supplementation or deficiency on microbiota composition.^11^ Under conditions of iron deficiency or supplementation, the relative abundance of certain bacteria is modified, with *Lactobacillaceae* and *Bifidobacteriaceae* being disfavored by iron supplementation.^11^ Overall, iron has a major impact on the microbiota, which extends beyond changes in composition to major modifications of the bacterial metabolite landscape, such as the production of short-chain fatty acids (SCFAs).^14^ There is no doubt the iron in the diet has an impact on the composition and activity of the microbiota, but whether iron stores in the body modify gut microbiota fitness has been less investigated thus far.

In the host, iron absorbed from the diet regulates the expression of numerous proteins involved in its homeostasis. For example, the levels of divalent metal ion transporter1 (Dmt1), which is responsible for transporting luminal iron into epithelial cells, and ferritin, an intracellular protein that stores iron, are inversely regulated by the amount of iron present in the body. Dmt1 levels increase under iron-restricted conditions, whereas those of ferritin decrease.^15–17^ Such iron-related regulation is orchestrated by an interplay of transcriptional and translational regulation mainly under the influence of the hormone hepcidin, intracellular iron regulatory proteins (IRPs), and the transcription factor Hif-2α.^17–21^ In accordance with the fact that the microbiota aids the global maturation of intestinal absorptive and secretory functions of the epithelium,^22–25^ microbial signals modulate both iron capture and storage.^26,27^ As with dietary iron, the Dmt1 and ferritin proteins are also inversely affected by the microbiota.^26,27^ Comparison of germfree (GF) and conventional specific pathogen free (SPF) mice under iron-replete conditions showed GF mice to exhibit lower ferritin levels and higher Dmt1 expression in the intestine.^26,27^ Various commensal strains, independently of their metabolic activity or level of implantation, influence the amount of intestinal ferritin.^26^ Under conditions of iron scarcity for a period of 14 days, the microbiota was shown to restrict iron absorption through the inhibition of Dmt1 expression, signifying competition between the intestinal microbes with the host for iron capture.^27^ The authors used an indirect assay based on co-incubation with HT-29 cells over-expressing Hif-2-α to ascertain the mechanisms responsible for the inhibitory effects of microbes on Dmt1 expression. In this model, reuterin and 1,3-diaminopropane (DAP), two bacterial metabolites, were able to inhibit Hif-2α.^27^ Thus, the gut microbial inhibition of intestinal HIF-2α limits host iron absorption.^27^ Moreover, in a mouse model of hemochromatosis, supplementation with DAP or a bacterial strain producing reuterin was able to decrease intestinal Dmt1 expression and subsequently reduce iron accumulation.^27^ Thus, the microbiota may help or hinder iron uptake and storage as a function of the physiological setting, kinetics, and diet. So far, the effects of a long term iron privation in GF and SPF have not been described.

It is well known that the duodenum and liver play crucial role in maintaining systemic iron homeostasis, but the majority of proteins involved in iron homeostasis, including Dmt1 and ferritin, are also expressed in the lung.^28^ Moreover, the effects of the intestinal microbiota may act at a distance in extra-digestive tissues, in particular, the lungs, through the so-called gut-lung axis.^29^ The interconnection between nutrition, the intestinal microbiota, and respiratory health has been extensively described for chronic respiratory diseases, viral and bacterial infections, and the physiological process of senescence.^6,33^ Although respiratory health and lung defense mechanisms are both linked to iron metabolism and intestinal microbiota activity,^32–34^ the dual effect of the microbiota and dietary iron has not been described in pulmonary iron regulation.^30,31^

We aimed to compare the effect of iron deficiency in GF and SPF mice over 60 days. Ferritin and Dmt1, which are both regulated by iron and the microbiota, were used as surrogate markers of intracellular iron stores and apical transport, respectively. We studied systemic iron parameters, ferritin and Dmt1 levels in the gut, liver, and lungs, modifications of the microbiota over time, and the involvement of Hif-2α. To understand the reciprocal interaction between host and microbiota, we evaluated whether iron in the diet can modify the microbiota and, conversely, whether iron stores in the body can modify gut fitness using a controlled mixture of microbes (*Enterococcus faecalis* + *Bacteroides thetaiotaomicron* + *Escherichia coli* + *Bifidobacterium longum*) that actively participate in maturation of the intestinal epithelium.^35^

## Materials and methods

### Animals and experimental design

**Figure 1.**
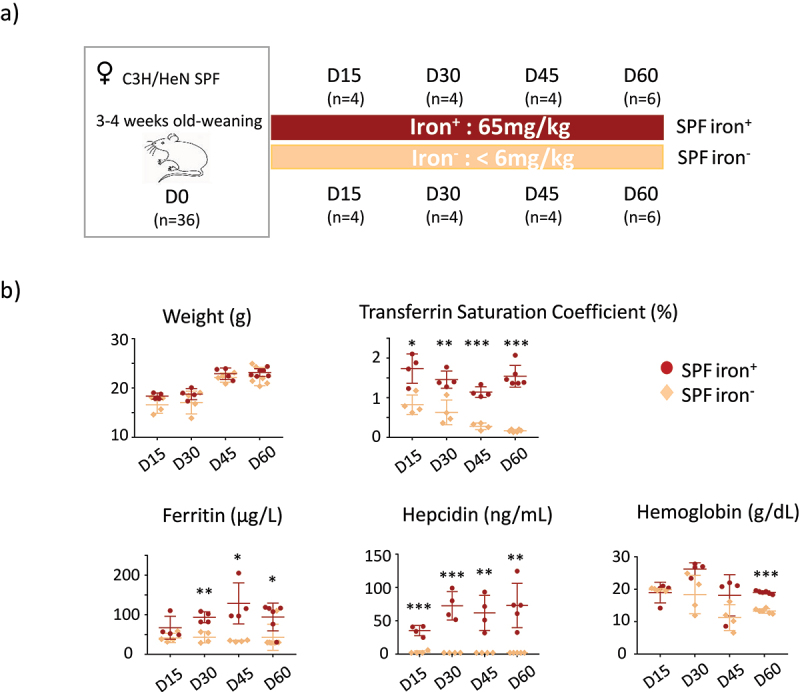
Protocol and systemic parameters for SPF mice.

The study was approved by the Animal Care and Use Committee of Jouy-en-Josas and the Ministry of Higher Education and Research (n° 20374– 2019060714134169V3). The protocol is based on a previous study using female mice with the same diets.^33^ Two groups of adult animals consisting of specific pathogen free (SPF) and germ free (GF) mice were used. Female SPF C3H/HeN mice (3 weeks old, *n* = 36) were purchased from Charles River Laboratories (Saint Germain Nuelles, France) and housed in the Rodent Experimental Infectiology Facility (IERP, INRAE Jouy-en-Josas, France). Female C3H/HeN GF (3 weeks old, *n* = 17) mice were bred from GF parents in the GF breeding facility of ANAXEM (germ-free animal facilities at INRAE, UMR1319 MICALIS, Jouy-en-Josas, France). The F0 generation was fed a standard diet containing 280 mg/kg iron (R03; Safe, Augy, France). All GF animals were housed in Trexler-type isolators (La Calhène, Vélizy, France). All SPF and GF animals were fed exactly the same diet (except for iron content) sterilized by 45 kGy gamma irradiation. The iron-deficient diet (iron^−^) contained <6 mg/kg iron (U8958 Version 0176) and the iron-containing diet (iron^+^) consisted of U8958P Version 0176 + 65 mg/kg iron (SAFE Nutrition, Augy, France)^36^ (Figure 1). The protocol was previously designed for the same duration for the GF and SPF groups, with data collected on days 15 (D15), 30 (D30), 45 (D45) and 60 (D60), but the iron-depleted diet could not be extended beyond 30 days for the GF mice without weight loss (Supplemental Figure S4).

(a) Schematic view of the protocol. All animals were 3 to 4 weeks old at the beginning of the protocol and received exactly the same diet except for iron content. Specific pathogen-free (SPF) female C3H/HeN mice were fed a diet containing 65 mg/kg or <6 mg/kg iron for 15 (D15), 30 (D30), 45 (D45), or 60 days (D60). The number of mice used for each time point is indicated in parentheses (n). Mice were euthanized to evaluate systemic, tissue, and cellular parameters. (b) Effects of an iron-deficient diet on the weight and plasma or blood parameters of SPF mice. The weight of the mice, plasma transferrin saturation coefficient, and ferritin (µg/L), hepcidin (ng/mL), and hemoglobin (g/dL) levels were measured on D15 (n = 4), D30 (n = 4), D45 (n = 4), and D60 (n = 6) for both the iron^−^ and iron^+^ groups. *p* values < .05 (*), < .005 (**), and < .0005 (***) indicate the significance of the differences between groups with and without iron by two-way ANOVA with Sidak’s multiple comparison test.

To obtain gnotobiotic mice, GF mice on iron-deficient (*n* = 3) and iron-rich (*n* = 2) diets were orally inoculated on D30 with 0.2 ml of a solution containing 10^6^ CFU of four strains: *Enterococcus faecalis* (strain X98), *Bacteroides thetaiotaomicron* (strain L55), *Escherichia coli* (strain CEC15), and *Bifidobacterium longum* (strain L74). Mice were euthanized four days after oral gavage. The gnotobiotic mice inoculated with the pure bacteria used here have been described elsewhere.^35^

Blood was collected by puncture of the orbital sinus using a glass pipette containing heparin. One hundred microliters of blood was placed in an EDTA-coated tube, maintained at 4°C, and used fresh for hemoglobin measurements the same day. The remaining blood sample was centrifuged two times at 4°C (2000 × g for 10 min) and 150 µL plasma was frozen at − 20°C until assayed.

After blood sampling, mice were euthanized by cervical dislocation and the cecal content and colon, duodenum, lung and liver samples were recovered and immediately frozen until analysis.

### Determination of the transferrin saturation coefficient and ferritin and hepcidin concentrations

Frozen plasma was used to determine ferritin (µg/L), transferrin (g/L), and iron (µmol/L) concentrations with an AU480 chemistry analyzer (Beckman Coulter/Olympus). The pourcentage coefficient of transferrin saturation was calculated using the formula: (plasma iron µmol/L)/(plasma transferrin (g/L) x 25). The concentration of hemoglobin (g/dL) was measured in fresh blood using an automated hemoglobin analyzer. The concentration of serum hepcidin was measured by LC-MS/MS, as described in.^37^ The threshold below which hepcidin could not be measured was 2 ng/mL.

### Determination of short chain fatty acid concentrations

Concentrations of SCFAs in the cecal content were determined after water-extraction (2 vol/wt) and protein precipitation using 10% (vol/vol) phosphotungstic acid (Sigma-Aldrich), as previously described.^38^ The internal standard 2-ethylbutyrate (Sigma-Aldrich) was used for quantification. Samples were analyzed in duplicate. Data were collected and the peaks integrated using OpenLab ChemStation C.01.06 software (Agilent, les Ulis, France). Data are expressed as µmol/g feces.

### 16S rRNA analysis

Genomic DNA was obtained from fecal samples at D1, thus 24 h after modification of the diet (*n* = 8, for each group), D30 (*n* = 3, for each group), and D60 (*n* = 5, for each group) using the QIAamp power fecal DNA kit (Zymo Research) and the DNA quantity determined using a TECAN Fluorometer (Qubit® dsDNA HS Assay Kit, Molecular Probes).

The V3-V4 hypervariable region of the 16S rRNA gene was amplified by PCR using the following primers: a forward 43-nuclotide fusion primer ^5′^**CTT TCC CTA CAC GAC GCT CTT CCG ATC T**AC GGR AGG CAG CAG^3′^ consisting of the 28-nt Illumina adapter (bold font) and the 14-nt broad range bacterial primer 343F and a reverse 47-nuclotide fusion ^5′^**GGA GTT CAG ACG TGT GCT CTT CCG ATC T**TA CCA GGG TAT CTA ATC CT^3′^ consisting of the 28-nt Illumina adapter (bold font) and the 19-nt broad range bacterial primer 784 R.

The PCR reactions were performed using 10 ng DNA, 0.5 µM primers, 0.2 mM dNTP, and 0.5 U of the DNA-free Taq-polymerase, MolTaq 16S DNA Polymerase (Molzym). The amplifications were carried out using the following profile: 1 cycle at 94°C for 60 s, followed by 30 cycles at 94°C for 60 s, 65°C for 60 s, 72°C for 60 s, and finishing with a step at 72°C for 10 min. PCR reactions were sent to the @Bridge platform (INRAE, Jouy-en-Josas) for sequencing by Illumina Miseq technology. Single multiplexing was performed using home-made 6-bp index sequences, which were added to R784 during a second PCR of 12 cycles using the forward primer (AATGATACGGCGACCACCGAGATTACACTCTTTCCCTACACGAC) and the reverse primer (CAAGCAGAAGACGGCATACGAGAT-index-GTGACTGGAGTTCAGACGTGT). The resulting PCR products were purified and loaded onto an Illumina MiSeq cartridge according to the manufacturer’s instructions. The quality of the run was checked internally using PhiX and the sequences then assigned to their sample using the previously integrated index sequences. High-quality filtered reads were further assembled and processed using the FROGS pipeline (Find Rapidly OTU with Galaxy Solution) to obtain the OTUs and their respective taxonomic assignment using Galaxy instance (https://migale.inra.fr/galaxy).^39^ For each dataset, > 97% of the paired-end sequences were assembled using at least 10-bp overlap between the forward and reverse sequences. The following successive steps involved de-noising and clustering of the sequences into OTUs using SWARM and chimera removal using VSEARCh. Then, cluster abundance was filtered at 0.005%. One hundred percent of clusters were affiliated with an OTU using the silva132 16S reference database and the RDP (Ribosomal Database Project) classifier taxonomic assignment procedure. The richness and composition of the bacterial community were computed using the Phyloseq package (v 1.19.1) in RStudio software.^40^ Within-sample community α-diversity was assessed by the observed diversity (i.e., the sum of unique OTUs per sample). Relative abundance at the phylum taxonomic level and representative OTUs are presented.

### PCR quantification of primobiota

*E. faecalis* (strain X98), *B. thetaiotaomicron* (strain L55), *E. coli* (strain CEC15), *B. longum* (strain L74) were quantified by real-time quantitative PCR analyses targeting the 16S rRNA genes, as described by Mayeur, et al..^41^

### Protein extraction for immunoblotting experiments

Total protein was prepared from frozen duodenum, liver, and lung samples as previously described.^42^ Briefly, tissues were crushed twice using a Tissue Lyser (Retsch) in lysis buffer (10 mM Tris, 20 mM NaCl, 5 mM MgCl_2_, 1 mM EDTA) supplemented with protease inhibitors (Roche) for 2 min. Then, tissues were further incubated for 60 min at 4°C with shaking in lysis buffer, centrifuged (20 min,10000 × g), and the supernatant recovered. To obtain the microsomal fraction (duodenum, liver, lung), tissues were crushed two times using a Tissue Lyser (Retsch) in stock buffer (0.25 M sucrose, 0.03 M L-Histidine, pH = 7.2, 100 μg/ml PMSF, 0.002 M EDTA, and protease inhibitors) for 2 min. The samples were then centrifuged 15 min at 6000 × g at 4°C to recover the post-nuclear supernatant, which was then transferred into polycarbonate tubes (Beckman). The samples were then centrifuged (80,000 × g, 45 min), the supernatant removed, and the microsomal fraction (pellet) resuspended in stock buffer. Total and microsomal proteins were measured using the Lowry assay.

### Immunoblotting experiments

Immunoblotting experiments were performed as previously described.^26^ The experimental conditions, antibody dilution, and quantity and treatment of protein extracts are described in Supplemental Data 1. Briefly, samples were separated by SDS-PAGE and transferred onto nitrocellulose membranes (Amersham Hybond) in Tris/glycine methanol buffer. Non-specific binding sites were saturated by incubation in 0.2 M Tris buffer (pH 7.5), 1.37 M NaCl (TBS) with 0.05% Tween 20 (TBST), and 5% (w/v) nonfat milk powder (Régilait). Membranes were incubated with all primary antibodies (listed in Supplemental Data, Table S1) overnight at 4°C on a rocking platform. Membranes were washed in TBST and probed with peroxidase-conjugated secondary antibodies at specific dilutions in TBST + 5% or 1% (w/v) nonfat milk powder (see Supplemental Table S1) for 2 h at room temperature on a rocking platform. Membranes were thoroughly washed and chemiluminescence detected using the Pierce^TM^ ECL2 Substrate kit, (Thermo Scientific). The chemiluminescence signals were detected using a ChemiDoc XRS (Bio-Rad) and analyzed using Image Lab software (Bio-Rad). Signal intensity is expressed as arbitrary values.

### Reverse transcription-quantitative PCR

Liver RNA was extracted using a mirVana^TM^ miRNA isolation kit (Invitrogen/ThermoFisher) Vilnius, Lithuania), as described in.^43^ RNA quality was determined using an RNA 6000 Nanoassay kit containing chips and the RNA integrity number (RIN) calculated using an Agilent 2100 Bioanalyzer. All tested RNA samples had a RIN above 8.5. RNA concentration A260/A280 and A260/A230 ratios were measured using a Nanodrop 2000 spectrophotometer. RNA samples were stored at −80^◦^C until use. Primers for cyclophilin (forward-^5’^ATGGCACTGGCGGCAGGTCC^3’^, reverse-^5’^TTGCCATTCCTGGACCCAAA^3’^) and *Hamp1* (forward-^5’^CCTATCTCCATCAACAGAT^3’^, reverse-^5’^TGCAACAGATACCACACTG^3’^) were used. Total RNA (3.5 µg) was converted to single-stranded complementary DNA (cDNA) using a High-Capacity cDNA Reverse Transcription Kit (Invitrogen/ThermoFisher Vilnius, Lithuania). Real-time PCR was performed using ROX SYBR^(r)^ Master Mix (TAKYON, Eurogentec Belgium) and mRNA levels were determined by the ΔΔCt method, with normalization relative to cyclophilin.

### Culture of the HT29 cell line for indirect assay to evalute the effect of bacteria on Hif 2α

We used an indirect assay based on co-incubation of HT-29 cells with microbial supernatants to monitor the action of bacteria on Hif-2α as described in.^27^ HT-29 cells were placed in a low-oxygen atmosphere (hypoxia) to boost the over-expression of Hif-2α^44^ and then co-incubated with supernatants from one of the bacteria strains used for primo-colonization of the GF mice. The HT29 cell line was used from passages 72–73 and were routinely grown in Dulbecco’s Modified Eagle Medium (DMEM, 1X) with GlutaMAX^TM^ (Gibco), supplemented with 10% (v/v) fetal calf serum (FCS) inactivated by incubation for 1 h at 56°C (Eurobio). Cells (1×10^5^ per mL) were grown for seven days in six-well tissue culture plates at 37°C in a 10% CO2:90% air atmosphere. At day 7, plates were incubated in DMEM 5% (v/v) heat-inactivated FCS and put in an anaerobic chamber containing a gas mix of 90% N_2_, 5% CO_2_ and 5% H_2_, without oxygen, for 6 h. An overnight anaerobic culture of *B. thetaiotaomicron* in LyBHi supplemented with 1 mg/mL cellobiose, 1 mg/mL maltose, and 0.5 mg/ml L-cysteine (4×10^8^CFU/mL) was centrifuged and 1 mL supernatant or LyBHi without bacteria (as a negative control) added for co-incubation in a final volume of 2 mL DMEM for 6 h in an anaerobic chamber. Cells were rinsed, scraped off, and immediately used for protein extraction following the protocol already described.

### Statistics

Nonparametric Mann–Whitney T-tests were used for comparisons between the groups with and without iron for D15, D30, D45, and D60. All data for weight, blood parameters, and short chain fatty acids were analyzed using 2-way ANOVA with Sidak’s multiple comparison test. The α-diversity was analyzed using T-tests. *p* values < .05 (indicated by *), < .005 (indicated by **), < .0005 (indicated by***) were considered statistically significant.

## Results

### Iron depletion over 60 days induces sequential and organ-specific effects in SPF mice

**Figure 2.**
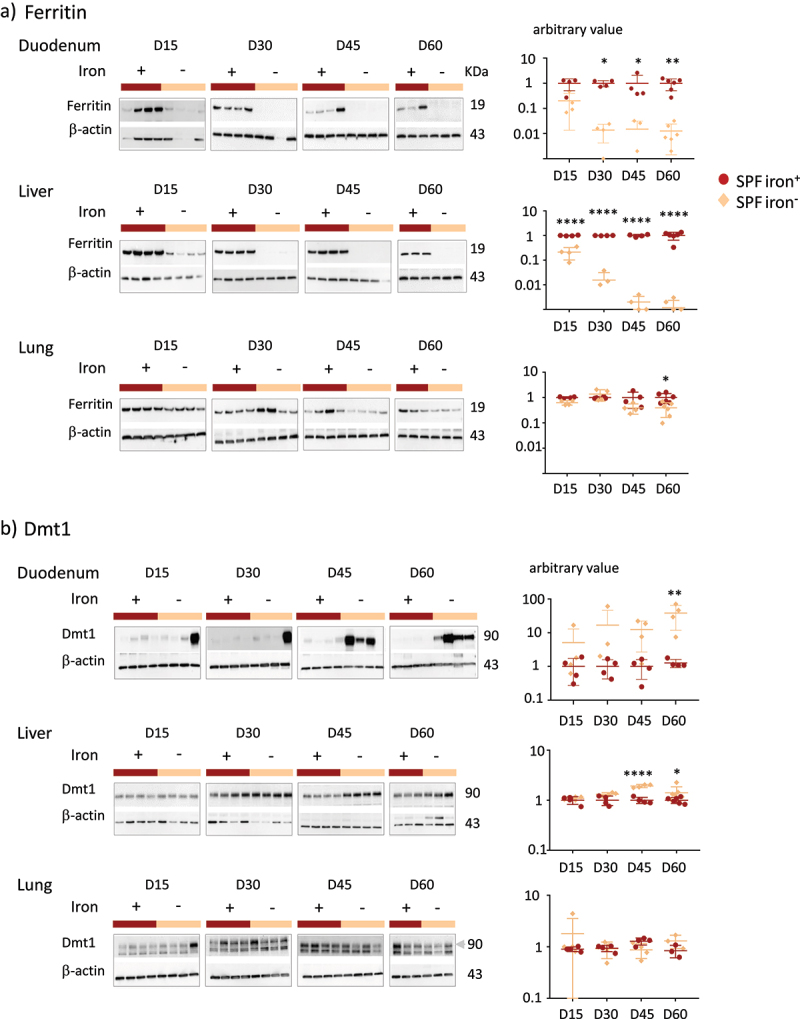
Effects of iron deprivation on tissue parameters of SPF mice.

We compared the effects of an iron-containing (iron^**+**^) and iron-depleted (iron^−^) diet for 15 (D15), 30 (D30), 45 (D45), and 60 (D60) days on mice harboring a conventional microbiota (SPF) (Figure 1a). The mice of both groups showed similar weight gain (Figure 1b). The saturation coefficient of transferrin significantly and progressively decreased from D15 with the iron^−^ diet. Plasma ferritin levels significantly decreased in the iron^−^ group from D30. Plasma hepcidin was below the threshold of detection for all iron^−^ groups, and we verified that the expression of the hepatic *Hamp1* gene (encoding hepcidin) was also low in mice fed the iron-depleted diet (Supplemental Data 2). Blood hemoglobin levels were significantly lower for the iron^−^ group on D60 than for the iron^**+**^ group. As previously described^36^ in female mice with similar age and comparable diet, it takes 60 days to induce iron deficiency anemia. These results validate the efficiency of the depletion of iron in the diet without modifying the weight gain of the mice. We then studied the effect of iron depletion on the tissues by quantifying ferritin (Figure 2a and Supplemental data 3) and Dmt1 (Figure 2b) protein levels in the duodenum, liver, and lungs. The amount of ferritin decreased in the duodenum and liver with the iron-depleted diet, whereas it remained unchanged (with some variability) in the lungs (Figure 2a). Dmt1 levels in the duodenum progressively increased, with notable inter-individual heterogeneity, in the absence of iron. The induction of Dmt1 expression was detected in one mouse on D15 and D30, in three mice on D45, and in all mice on D60. The longer iron privation persisted, the less inter-individual variation was observed in the duodenum. Dmt1 levels were less affected by iron in the extra-gut tissues. In the liver, they increased slightly by D45 and D60 in the iron^−^ conditions and remained unchanged in the lungs.

Protein levels were assessed by western-blot analysis on D15 (*n* = 4), D30 (*n* = 4), D45 (*n* = 4), and D60 (*n* = 6) for the duodenum, liver, and lungs of SPF mice in the iron^+^ and iron^−^ groups. A representative western blot is shown for each condition. β-actin was used as a loading control. a) Western blots (representative membranes) and quantification (graphs) of ferritin in total protein extracts. All values were normalized relative to the mean ferritin signal at D15 for mice fed the iron^+^ diet. b) Western blots (representative membranes) and quantification of Dmt1 in membrane protein extracts. As described by Yanatori et al., ^45^DMT1 appears on the western blot as 90–116 kDa smear. The arrow indicates the upper band used for quantification of DMT1 in lung. All values were normalized relative to the mean of Dmt1 at D15 for mice fed the iron^+^ diet. *P* values < .05 (*), < .005 (**), <.00001 (****) indicate the statistical difference between the iron^−^ and iron^+^ groups for each time point by two-way ANOVA with Sidak’s multiple comparison test.

Overall, ferritin and Dmt1 levels were inversely and sequentially modified by iron privation, in agreement with the previous observations.^15–17^ The mobilization of intestinal and hepatic iron from ferritin was rapid, whereas Dmt1 levels increased late in the duodenum of all mice (Figure 2a,b). In the lungs, the amount of ferritin and Dmt1 remained constant up to 45 days of iron privation (Figure 2a,b).

### Germ-free mice have low intestinal ferritin and are poorly resistant to iron-deficient diet

**Figure 3.**
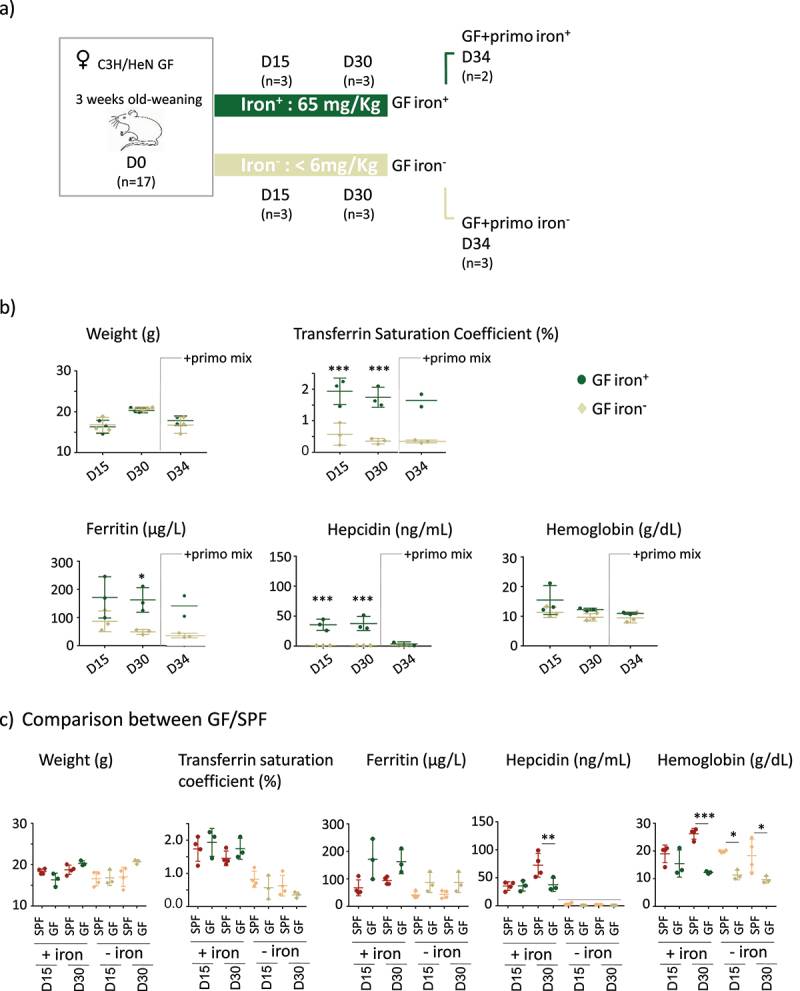
Protocol and parameters for GF mice.

**Figure 4.**
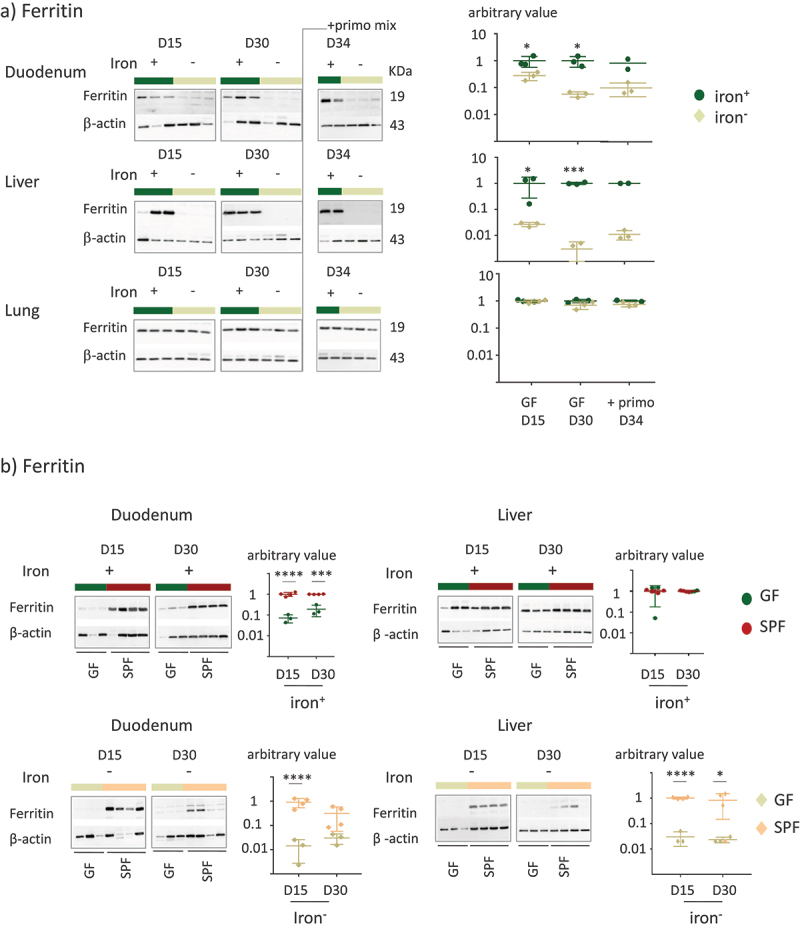
Effect of iron privation on ferritin in GF and SPF mice.

In an initial experiment, we replicated the protocol used on the SPF mice on GF mice, using time points of 15, 30, 45, and 60 days. However, prolonged iron depletion in GF mice for over 30 days resulted in a reduction in weight gain (Supplemental Figure S4). We thus limited our observations of the GF mice to 15 and 30 days to prevent confounding the effects of iron depletion with those related to restricted growth (Figure 3a). At D30, we inoculated the mice with a mixture of primo-colonizing bacteria (+primo) to establish whether iron depletion modifies gut fitness through colonization by the microbiota (Figure 3a). Mice inoculated with primo-colonizing bacteria were studied not more than four days after the gavage (D34) to avoid a prolonged protocol that was deleterious for the growth of the GF mice (Supplemental Figure S4). Mice in both the iron^+^ and iron^−^ groups showed similar weight gain (Figure 3b). Transferrin saturation and plasma ferritin and hepcidin protein levels (as well as corresponding mRNA, Supplemental Data 2) were significantly lower in GF mice receiving the iron-depleted diet, whereas the content of iron in the diet did not affect the level of hemoglobin (Figure 3b). We did not observe any change in any of these parameters after inoculation with the primo-mix (GF+primo), except for plasma hepcidin levels, which dropped by D34 in the iron^+^ group (Figure 3b). We plotted the data obtained from both the GF and SPF mice on the same graph to facilitate their comparison (Figure 3c). Overall comparison between the GF and SPF groups receiving the iron^+^ diet showed the GF status to be associated with lower hemoglobin and hepcidin levels on D30 (Figure 3c). GF mice receiving the iron^−^ diet showed significantly lower hemoglobin levels at D15 and D30 than SPF mice (Figure 3c). We examined the effect of iron depletion on ferritin levels in the duodenum, liver, and lungs of GF and GF+primo mice. Ferritin levels decreased over time in the duodenum and liver in GF mice receiving the iron^−^ diet, whereas it remained stable in the lungs (Figure 4a). Mice in the GF iron^−^ group showed greater depletion of ferritin in the liver than in the duodenum, suggesting that the hepatic stock of iron was preferentially mobilized in this condition. With an iron-containing diet (Figure 4b, upper panels), ferritin levels in the duodenum in GF mice were lower than those in that of SPF mice, showing that GF mice had lower intestinal stores of iron but similar hepatic stores. The privation of iron up to 30 days led to drastic mobilization of ferritin in the duodenum and liver in GF mice, whereas the SPF mice had greater stores (Figure 4b, lower panels).

a) Schematic view of the protocol. Female germ-free (GF) C3H/HeN mice were fed a diet containing 65 mg/kg iron or <6 mg/kg for 15 (D15) or 30 days (D30) because a longer period of iron privation led to weight loss (Supplemental data 4). On day 30, GF mice were inoculated with a mix of four primo-colonizing bacteria (GF+primo) by oral gavage. The primo-colonizing bacteria consisted of *E. faecalis* (strain X98), *B. thetaiotaomicron* (strain L55), *E. coli* (strain CEC15), and *B. longum* (strain L74). Mice with the primo-mix were euthanized four days (D34) after inoculation. Mice were euthanized to evaluate the systemic, tissue, and cellular parameters. The number of mice used for each time point are indicated in parentheses (n). b) Effects of an iron-poor diet on the weight and plasma parameters of GF and GF+primo mice. The weight (g), transferrin saturation coefficient (%), and ferritin (µg/L), hepcidin (ng/mL), and hemoglobin (g/dL) levels were determined on D15 (*n* = 3 iron^+^, and *n* = 3, iron^−^), D30 (*n* = 3, iron^+^ and *n* = 3, iron^−^), and D34 (*n* = 2, iron^+^ and *n* = 3, iron^−^) after the beginning of the intervention. *P* values < .05 (*), < .005 (**), and < .0005 (***) indicate the statistical difference between the groups fed diets with and without iron by two-way ANOVA with Sidak’s multiple comparison test (the D34 group was excluded because *n* = 2). c) Compilation of the parameters obtained for GF and SPF mice at D15 and D30 for the iron^+^ and iron^−^ conditions. The line indicates the threshold of detection of serum hepcidin (2 ng/mL). *P* values < .05 (*), < .005 (**), and < .0005 (***) indicate the statistical difference between the GF and SPF groups for each condition by two-way ANOVA with Sidak’s multiple comparison test.

a) The amount of ferritin in the tissues of GF and GF+primo mice was studied by western blotting on D15 (*n* = 3), D30 (*n* = 3), and D34 (*n* = 2) for the iron^+^ group and on D15 (*n* = 3), D30 (*n* = 3), and D34 (*n* = 3) for the iron^−^ group. Western blots and quantification of ferritin in total protein extracts from the duodenum, liver, and lungs of GF and GF+primo mice. A representative western blot is shown for each condition. β-actin was used as a loading control. All values are normalized relative to the mean of ferritin on D15 for mice fed the iron^+^ diet. *P* values < .05 (*) and < .0005 (***) indicate the statistical difference between the iron^−^ and iron^+^ groups for each time point by two-way ANOVA with Sidak’s multiple comparison test. b) Duodenum and liver proteins from GF and SPF were loaded on the same gel to allow the comparison of ferritin between groups over time (*n* = 3 for GF and *n* = 4 for all SPF groups). All values are normalized relative to the mean of ferritin on D15 for SPF mice fed the iron^**+**^ diet (upper panel) and D15 for SPF mice fed the with iron^−^ diet (lower panel). *P* values < .05 (*) and < .0001 (****) indicate the statistical difference between GF and SPF mice for each time point by 2 two-way ANOVA with Sidak’s multiple comparison test.

### Dietary iron and microbiota inhibit intestinal Dmt1

**Figure 5.**
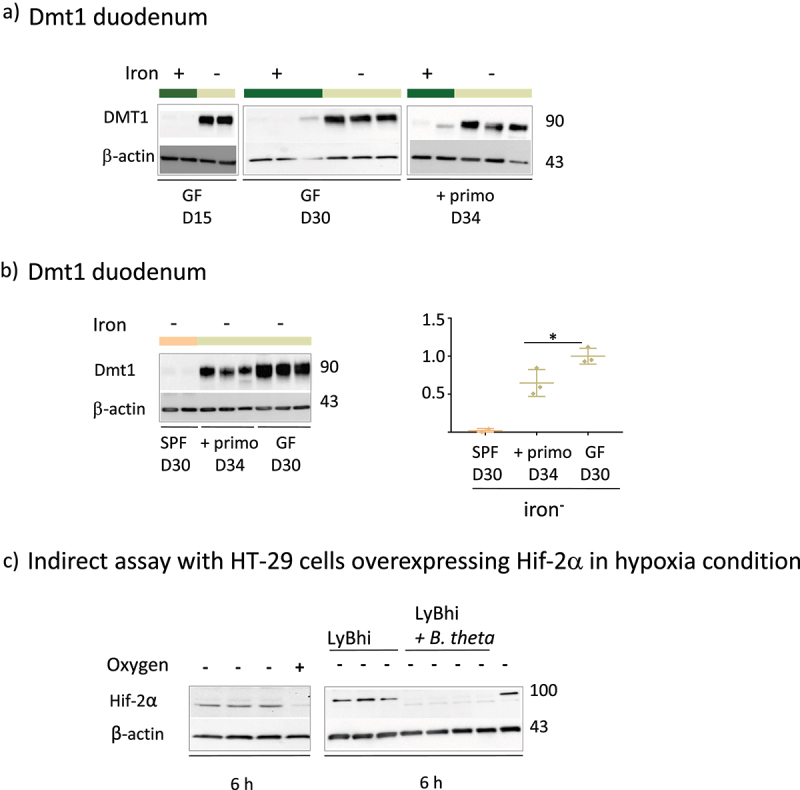
Effect of iron privation and the microbiota on Dmt1 expression and Effect of *B. thetaiotaomicron* supernatant on Hif-2α protein.

The iron-poor diet increased Dmt1 levels in the duodenum of GF mice at D15 and D30 (Figure 5a). Comparison of the three groups (Figure 5b) showed that Dmt1 levels decreased in the presence of the microbiota (compare the SPF *vs* GF+primo *vs* GF groups). The reduction in Dmt1 levels was greater as a function of the complexity of the microbiota and the length of time it was present (compare the SPF *vs* GF+primo groups). We assessed the involvement of HIF-2α to examine the mechanisms responsible for the decrease in Dmt1 levels in the presence of the microbiota (Figure 5c). Given the low level of expression of HIF-2α in the animals, we used an indirect assay based on Hif-2α over-expressing HT-29 cells according to established protocols.^27,44^ We cultured HT-29 cells under hypoxic conditions (oxygen-) with culture medium without (LyBhi) and with bacteria (LyBhi+theta) (Figure 5c). The expression of Hif-2α was boosted when the HT-29 cells were maintained under anaerobic conditions for 6 h (Figure 5c, left panel). The hypoxia-induced level of Hif-2α was repressed when the cells were co-incubated with a culture supernatant of *B. thetaiotaomicron*, one of the bacteria of the primo-mix (Figure 5c, right panel).

a) Western blots and quantification of Dmt1 in duodenum membrane protein extracts on D15 (n = 2), D30 (n = 3), and D34 (n = 2) for mice in the iron^+^ group and D15 (n = 2), D30 (n = 3), and D34 (n = 3) for mice in the iron^−^ group. A representative western blot is shown for each condition. β-actin was used as a loading control. b) Western blot and quantification of Dmt1 in duodenum membrane protein extracts from SPF D30 (n = 2), GF+primo D34 (n = 3), and GF D30 (n = 3) mice fed the iron^−^ diet. All values are normalized relative to the mean of Dmt1 on D30 for GF mice fed the iron^−^ diet. *p* values < .05 (*) indicate the statistical difference between the GF and GF+primo groups by the nonparametric Mann–Whitney T-test. c) Effect of *B.* *thetaiotaomicron* supernatants on Hif-2α protein levels in HT29 cells. HT29 cells were incubated 6 h in the absence (-) or presence (+) of oxygen and then co-incubated with LyBhi or LyBhi mixed with an equal amount of a supernatant of an over-night culture of *B.* *thetaiotaomicron*. A representative western blot of HIF-2α and β-actin proteins is shown.

### Iron privation led to reduced activity and diversity of microbiota, but favored certain commensals

**Figure 6.**
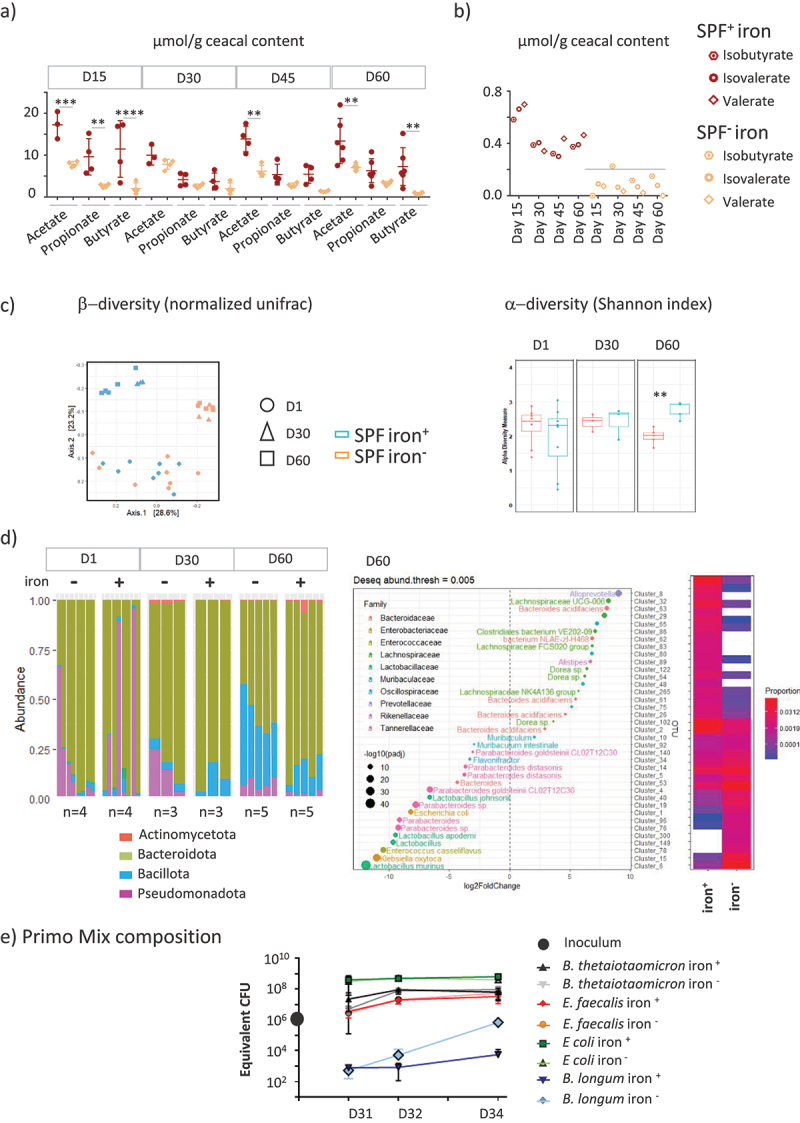
Reciprocal effects of iron deprivation on microbiota.

The iron content in the diet modified the fermentative activity of the cecal microbiota over time, as measured by changes in the levels of major SCFAs (Figure 6a) and less abundant branched SCFAs (Figure 6b) in the cecum of SPF mice. The initial 15 days without iron led to significantly lower production of acetate, propionate, and butyrate. The differences in SCFA production between the two groups were no longer observable by D30. After iron privation for 45 and 60 days, less acetate and butyrate were produced in the iron^−^ group, whereas the propionate levels remained equivalent in the two groups. Branched-SCFAs decreased to almost under the level of detection in all iron-depleted groups (Figure 6b). The microbiota β-diversity, determined by the normalized UNIFRAC index, evolved over time between D1, D30, and D60 (Figure 6c), with differences between the iron^−^ and iron^+^ groups becoming observable from D30. Iron privation for 60 days significantly reduced the α-diversity (Figure 6c), with *Pseudomonadota* and *Bacillota*, in particular, certain species of *Lactobacillales* being favored at the expense of *Bacteroidota* (Figure 6d). We examined whether iron content in the body could modify the fitness of commensals bacteria, by following the kinetics of colonization one (D31), two (D32), and four days (D34) after gavage of the four primo-colonizing strains in the iron-poor and iron-rich conditions (Figure 6e). At D31, the proportion of each bacterial strain was *E. coli* > *B. thetaiotaomicron* = *E. faecalis* > *B. longum* for both groups. Four days after gavage (D34), the hierarchy between the strains was maintained, but the *B. longum* strain became 2 log more abundant in the iron^−^ group (Figure 6e). At D34, the levels of acetate, which was the only detectable SCFA produced, were not significantly different between the iron^+^ (10.8 ± 0.4 mM, *n* = 2) and iron^−^ (13.2 ± 4 mM, *n* = 3) groups.

a) Acetate, proprionate, butyrate, and b) minor short chain fatty acids were measured in the cecum of SPF mice at D15, D30, D45, and D60 from the iron^−^ and iron^+^ groups. The line indicates the threshold of detection (under 0.4 µmol/g). *p* values < .005 (**), < .0005 (***), and < .0001 (****) indicate the statistical difference between groups fed diets with and without iron by two-way ANOVA with Sidak’s multiple comparison test. c) diversity index of the microbiota in SPF mice receiving the two diets for 1, 30, and 60 days.Left panel: β-diversity by the normalized UNIFRAC index. Right panel: box plot showing α-diversity using the Shannon index. *p* values < .005 (**) indicate the statistical difference between groups fed diets with and without iron by two-way ANOVA with Sidak’s multiple comparison test. d) Microbiota composition based on 16 S sequences from fecal samples of mice from the iron^−^ and iron^+^ groups at different time points. Left panel: phylum level. Right panel: different operational taxonomic units expressed on D60 as a function of diet. e) At D30, GF mice were orally inoculated with a mixture (∙) containing 10^6^ CFU of *Enterococcus faecalis* (strain X98), *Bacteroides thetaiotaomicron* (strain L55), *Escherichia coli* (strain CEC15), and *Bifidobacterium longum* (strain L74). Each strain was equivalently represented in the inoculum. Quantification by real-time PCR of each primo-colonizing strain one day (D31), two days (D32), and four days (D34) after inoculation for the iron^+^ (n = 2) and iron^−^ (n = 2) groups.

## Discussion

The objective of this study was to compare the effect of iron deficiency in GF and SPF mice to evaluate over time the intricate and reciprocal effects of dietary iron, the microbiota, and the iron metabolism of the host. While acknowledging the small sample size of some gnotobiotic groups, we show that the microbiota and the host organism form a dynamic duo that transitions from cooperation to competition depending on the level of iron in the diet.

The following findings confirm previously reported conclusions:^26,27^ a) Microbiota-mediated inhibition of host iron absorption via the HIF2 pathway under iron depletion b) Abundance of *Lactobacillaceae* under dietary iron deficiency c) Host ferritin accumulation by gut microbiota. Here, the long duration of iron privation highlights the progressive and organ-specific kinetics of adaptation (see results with DMT1, microbiota activity, hepatic and intestinal stores). We also show for the first time that an iron-depleted body (due to an iron-poor diet) affects the fitness of the gut and the dynamics of the colonization process with a controlled mix of microbes.

In SPF mice, 60 days of iron depletion resulted in a drop in serum iron marker levels, poor SCFA production, a less diverse microbiota, sub-dominance of *Bacillota*, especially *Lactobacillales*, and lower ferritin and higher Dmt1 levels in the duodenum and liver. These parameters all evolved over the time of privation, consistent with previous data describing the progressive adaption of the host to cope with iron privation.^46^ In GF mice, we observed that 1) an iron-depleted diet could not be extended beyond 30 days without weight loss, 2) as they aged they showed less blood hemoglobin than SPF mice, even with a diet rich in iron (Figure 3c), 3) the pool of duodenum ferritin was low, possibly explaining the rapid mobilization of their hepatic stores in situations of iron privation, and 4) a strong induction of Dmt1 expression due to iron restriction.

GF mice receiving the iron^−^ diet showed significantly lower hemoglobin levels and this finding is in contrast with previous report.^27^ This discrepancy may be related to different genetic background of mice (C3H/HeN versus C57BL/6J) and to differences in initial hepatic and intestinal iron stores in the mice at the beginning of the protocol. In accordance with the results of the two previous results,^26,27^ in the presence of the microbiota, the amount of intestinal ferritin increased when the diet contained an adequate amount of iron, which may aid resistance against iron privation. Our results are also in accordance with historic observations showing increased iron retention in the body in the presence of microbiota during conventionalization of recipient GF rats.^47^ Given the various roles of ferritin in metabolic disorders, inflammation, infection, and cancer, it is likely that microbiota-linked regulation of ferritin is determinant in the intricate relationship between iron-microbiota-ferritin and pathologies^48^ and may promote immune tolerance.^49^

In the situation of iron privation, Dmt1 production was rapidly and massively induced in GF mice, which have only small stores of endogenous iron. In SPF mice, the induction of Dmt1 production occurred later in all mice and its amplitude was smaller. The use of gnotobiotic mice (GF+primo mix *vs* GF) confirmed that the presence of microbes reduces Dmt1 levels and illustrates competition with the host in situations of iron scarcity, as already suggested by previous observations.^26,27^ We also observed a similar pattern of expression for the protein DcytB, which is a membrane protein that reduces ferric iron to ferrous iron, a preliminary step essential for its absorption by the duodenal protein Dmt1 (Supplemental Data 5). Such microbiota linked-lowering of Dmt1 levels appears to be mainly driven by HIF-2α in particular in response to yet unidentified molecules produced by *B. thetaiotaomicron* (Figure 5). Thus, the microbial-linked extinction of the transporter Dmt1 is at least partially sustained by the reduction of HIF-2α, which is a major molecular actor that governs its expression.^50^

Our results show that the microbiota plays a versatile role depending on the amount of available iron. Under conditions of sufficient iron, the microbiota favors intestinal storage. On the other hand, under conditions of iron deficiency, the microbiota inhibits the expression of the luminal intestinal DMT1 transporter, illustrating competition with the host. The fact that the microbiota favors resistance against iron privation by helping the host to store iron through higher amounts of ferritin can be considered as cooperation between commensals and the host. The gut microbiota also plays a crucial role in improving the bioavailability of iron for the host by enhancing the production of SCFA.^51^ However, in the case of iron privation, commensals induce a decrease in the levels of the Hif-2α-dependent Dmt1 transporter, reflecting competition between the microbiota and host. Thus, the effect of the microbiota on iron-related metabolism is to detect and react to temporal signals to sustain cooperation or competition with the host depending on iron availability.

This is the first demonstration that the composition and activity of the intestinal microbiota dynamically adapts to iron deficiency and that, conversely, the ability of bacteria to colonize the gut is altered when the body’s iron reserves are depleted. The depletion of iron in the diet modified the activity, diversity, and composition of the microbiota over time. The massive reduction of SCFA synthesis at 15 days was less strong with longer iron depletion. The fluctuation of microbiota that we observed over time could explain the difficulty in observing clear trends of the iron-related evolution of the microbiota in compiling different studies. The pattern of changes is linked to the initial microbiota, the duration of treatment, and the form of iron used.^11^ After 60 days of iron privation, the α-diversity and production of acetate and butyrate were reduced, and *Bacillota* became more dominant. Iron privation also had an impact on the time course of colonization of a mixture of bacterial strains. The inoculation of a mixture of four strains showed progressive colonization of the recipient gut by the bacteria, with a 2 log increase between D31 and D34 (Figure 6e). Among these four strains, *E.coli* was the most abundant at D31 and it remained the most abundant strain until D34. The increasing number of bacteria and the predominance of *Enterobacteriacea* mimic the kinetics of implantation of the microbiota described at birth.^24,52^ *B. longum* was the only strain that thrived in an iron-depleted environment, as it was 2 log more abundant at D34 in the iron^−^ group. All four strains were cultivated in a culture medium containing iron before inoculation. Thus, it is likely that the iron stores of the bacteria were replenished and could be mobilized to ensure their implantation. In our experimental setting, we cannot exclude that the supremacy of *B. longum* is linked to more efficient storing of iron. However, the advantage of *B. longum* at D34 in the iron-depleted environment (Figure 6e) is consistent with observations made in Human cohorts, in which the *Bifidobacterium* group is prevalent in anemic individuals^53^ and iron deficiency leads to a reduction in butyrate-producing anaerobic bacteria.^54^ *Bifidobacterium* strains are also genetically equipped to resist low-iron conditions.^53,55^ The installation of a simplified microbiota in recipient GF mice is modulated by iron, with a selective advantage for *Bifidobacterium* in iron-depleted conditions.

For many pathogens, virulence is linked to their effectiveness in acquiring iron and maintaining iron levels. Thus, iron overload is associated with an increased risk of infection^56^ and iron fortification can favor pathogen abundance.^57^ The host limits iron availability to invading microbes as an efficient mechanism of defense known as nutritional immunity.^58,59^ In the case of beneficial microbes, certain probiotic strains are involved in a positive effect on iron absorption, although there is not yet clear evidence in humans.^60^ P-hydroxyphenyl lactic acid secreted by the probiotic microbe *L. fermentum* reduces ferric iron to ferrous iron, thus making it bioavailable to the host.^61^ Probiotics may also help the host by competing for iron sequestration with pathogens. The *Escherichia coli* strain Nissle 1917 can reduce *Salmonella enterica* serovar Typhimurium colonization in mouse models of colitis and limits chronic persistent infection by iron acquisition.^62^ Thus, the interplay for iron metabolism between microbes and the host is adjustable; commensals can act both as cooperative partners and competitors, whereas pathogens are more clearly competitors and probiotics directly help the host by favoring iron bioavailability or indirectly by competing with pathogens for iron. The functional continuum between pathogens, commensals, and probiotics leads to fine-tuned host-iron-microbe crosstalk, ranging from competition to cooperation in the gut.

We also show that lung iron stores are spared in a low-iron diet, as if this organ were protected. Iron storage, import, and export concern all organs, which have specific iron requirements, but many of these differences are still poorly documented for extra-digestive epithelia, such as in the lungs. Neither storage with ferritin nor levels of Dmt1 were affected by iron deficiency in the lung, either in SPF or GF mice, suggesting that the lungs are protected from iron deficiency (Figures 2 and 4). Thus, when iron becomes limiting, the iron distribution to tissues becomes prioritized, with the lungs being particularly well protected. In a murine model in which hepcidin production is impaired (Hepc KO mice), leading to iron overload, an increase in plasma iron levels, a drop in Dmt1 levels, and the accumulation of L-ferritin were found in the lungs.^63^ In the lungs of iron over-loaded mice, iron is taken up by pulmonary cells and further passed to macrophages of the alveoli, where it is safely stored. We did not quantify iron storage in alveolar macrophages during the iron-depleted diet, but it is possible that they may provide iron and serve as a reservoir for rescue. As observed in the iron-overload model, we provide evidence of the resistance of the lungs to maintain iron stores in the situation of an iron-depleted diet, independently of the microbiota.

In conclusion, iron metabolism, storage, and transport are mainly governed by the amount of iron in the diet and the microbiota in the splanchnic area but not the lungs. Reciprocally, the amount of iron in the diet and iron stores in the body dynamically modulate the composition and activity of the microbiota and gut fitness. With a diet containing an adequate amount of iron, the presence of the microbiota increases iron storage in the body and favors resistance against privation. If the diet becomes iron deficient, commensals affect the duodenum expression of the iron transporter Dmt1 and thus impair iron transport, mainly through the Hif-2α signaling pathway.

## Supplementary Material

Supplemental Material

## Data avaibility statement

The authors confirm that the data supporting the data sets are available in the “DATA INRAE” repository platform accessible through doi.org/10.57745/8ABKFG. The data and calculations deposited are all weight and iron parameters of mice, qPCR screen shot for Hamp1 quantification, short chain fatty acids, microbiota sequences, raw membranes of all westerns blots.
