# Supplementary material for: The microbiota and the host organism switch between cooperation and competition based on dietary iron levels
Source: Gut Microbes. 2024 Jun 27;16(1):2361660. doi: 10.1080/19490976.2024.2361660 (PMC11212566; doi:10.1080/19490976.2024.2361660)
Supplement: Supplemental Material [file KGMI_A_2361660_SM4580.zip › all_supplemental_figures.docx]

Supplemental data 1: Experimental conditions of western blotting and list of antibodies Total Proteins from Colon, Duodenum, Lung, Liver (extraction buffer lysed with Tissue Lyser)

Membrane Proteins from Duodenum, Liver, Lung (extraction buffer : L-Histidine lysed with Tissue Lyser)

|  | **Loading** | **Heat denaturation**  **before loading** | **Antibody 1** | **Antibody 2** | **Loading**  **Control** |
| --- | --- | --- | --- | --- | --- |
| **L-Ferritin** | Total extract  (15µg) | Yes | SAB2500431  Sigma-aldrich Dilution 1/500 | Rabbit Anti Goat  40 15 15  Calbiochem Dilution 1/10000 | β-actin  A5316  Sigma-Aldrich |
| **Dcytb** | Membrane fraction (30µg) | Yes | Dcytb11-A Alpha Diagnostic Dilution 1/500 | Anti-rabbit 711-035-152  Jackson Immuno Research Dilution 1/10000 | β-actin (Ascites fluid A5316  Sigma-Aldrich |
| **DMT1** | Membrane fraction (20µg) | No | From François Canonne-Hergaux Dilution 1/500 | Anti-rabbit 711-035-152  Jackson Immuno Research Dilution 1/10000 | β-actin A5316  Sigma-Aldrich |
| **HIF2 α** | Total extract  (30µg) | Yes | NB 100-122 NOVUS  Biologicals  Dilution 1/500 | Anti-rabbit 711-035-152  Jackson Immuno Research Dilution 1/10000 | β-actin A5316  Sigma-Aldrich |

Supplemental data 2

*Hamp1* liver expression (coding hepcidin) by real-time RT-PCR analysis in different groups with or without iron. For each group, the number of mice (n) is indicated. The values were expressed as 2-ΔΔCt, with normalization relative to cyclophilin. Significant results between the two groups per day by t-test.

| **2-ΔΔCt** | **Hamp1 (liver)** |
| --- | --- |
| SPF iron+ (n=3) *vs* SPF iron– (n=3) Day 30 | 17000 |
| GF iron+ (n=3) *vs* GF iron– (n=3) Day 30 | 13000 |
| Primo Mix ^iron+^ ^(n=2)^ *vs* Primo Mix ^iron^– (n=3) Day 34 | 3000 |

Supplemental data 3. Mean weight (g) of germ free mice (n=4, at each point) receiving iron containing diet or iron poor diet for 15, 30, 45 and 60 days. P values < 0.05 (*), < 0.005 (**), indicated the comparisons between groups with and without iron by 2-way ANOVA with Sidak’s multiple comparison test.


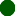
i r o n +
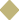
 i r o n -


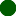

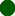

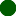

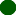

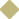

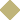

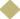

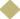


**2 4**

**2 2**

**2 0**

*

**

**1 8**

**1 6**

**1 4**

**1 2**

**w e i g h t ( g )**

D15

D30

D45

D60

Supplemental data 4

The amount of Dcytb protein were studied by western blot at 30 or 34 day in duodenum in SPF, GF+primo and GF in iron**-** and iron+ groups. A representative membrane of western blot was shown for each condition. β-actin was used a loading control. Membrane protein extracts were used, (n) indicated the number of mice used.

Duodenum

Iron

Dcytb

β-actin

D30 D34 D30

- - -


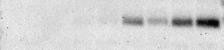

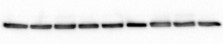


D30 D34 D30

+ + +


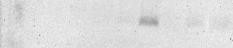

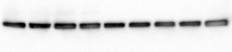


KDa 28

43

SPF (n=3)

+ primo (n=3)

GF (n=3)

SPF (n=4)

+ primo (n=2)

GF (n=3)
